# Supplementary material for: Conductance-based dendrites perform Bayes-optimal cue integration
Source: PLoS Comput Biol. 2024 Jun 12;20(6):e1012047. doi: 10.1371/journal.pcbi.1012047 (PMC11168673; doi:10.1371/journal.pcbi.1012047)
Supplement: S1 Text — 1. Definitions. 2. Derivation of the somatic potential distribution. 3. Derivation of membrane potential dynamics. 4. Derivation of weight dynamics. 5. Unreliable dendritic inputs are assigned small synaptic strengths. 6. Dendritic parameters. (PDF) [file pcbi.1012047.s001.pdf]

Supplementary material for
Conductance-based dendrites perform Bayes-optimal cue integration

Jakob Jordan[1,2]*, João Sacramento[1,3], Willem A.M. Wybo[1,4],
Mihai A. Petrovici[1†] & Walter Senn[1†]

[1]Department of Physiology, University of Bern, Bern, Switzerland

[2]Electrical Engineering, Yale University, New Haven, CT, United States

[3]Institute of Neuroinformatics, UZH / ETH Zurich, Zurich, Switzerland

[4]Institute of Neuroscience and Medicine, Forschungszentrum Jülich, Jülich, Germany

March 12, 2024

Supplements

---

*Correspondence: jakob.jordan@unibe.ch

†Joint senior authorship.

1 Definitions

The following definitions are used throughout the supplementary material and main manuscript:

$$
\begin{aligned}
u_{\mathrm{s}} =& \text{somatic membrane potential} \\
\lambda_{\mathrm{e}} =& \text{neuronal exploration parameter} \\
W_i^{\mathrm{E/I}} =& \text{excitatory/inhibitory synaptic weights onto dendrite } i \\
r_i =& \text{presynaptic rates} \\
g_i^{\mathrm{L}} =& \text{leak conductance on dendrite } i \\
g_i^{\mathrm{E/I}} =& W_i^{\mathrm{E/I}} r, \text{excitatory/inhibitory conductance on dendrite } i \\
E^{\mathrm{L/E/I}} =& \text{leak/excitatory/inhibitory reversal potential} \\
g_0 =& \text{prior conductance} \\
E_0 =& \text{prior potential} \\
g_i^{\mathrm{d}} =& g_i^{\mathrm{E}} + g_i^{\mathrm{I}} + g_i^{\mathrm{L}} \text{ isolated dendritic conductance} \\
E_i^{\mathrm{d}} =& \frac{g_i^{\mathrm{E}} E^{\mathrm{E}} + g_i^{\mathrm{I}} E^{\mathrm{I}} + g_i^{\mathrm{L}} E^{\mathrm{L}}}{g_i^{\mathrm{E}} + g_i^{\mathrm{I}} + g_i^{\mathrm{L}}} \text{ dendritic reversal potential} \\
g_i^{\mathrm{sd}} =& \text{dendro-somatic coupling conductance} \\
g_i^{\mathrm{ds}} =& \text{somato-dendritic coupling conductance} \\
\alpha_i^{\mathrm{sd}} =& \frac{g_i^{\mathrm{sd}}}{g_i^{\mathrm{ds}} + g_i^{\mathrm{d}}} \text{ dendro-somatic coupling factor} \\
\alpha_i^{\mathrm{ds}} =& \frac{g_i^{\mathrm{ds}}}{g_i^{\mathrm{ds}} + g_i^{\mathrm{d}}} \text{ somato-dendritic coupling factor} \\
\bar{g}_{\mathrm{s}} =& g_0 + \sum_{i=1}^{D} \alpha_i^{\mathrm{sd}} g_i^{\mathrm{d}} \text{ total somatic conductance} \\
\bar{E}_{\mathrm{s}} =& \frac{1}{\bar{g}_{\mathrm{s}}} \left(g_0 E_0 + \sum_{i=1}^{D} \alpha_i^{\mathrm{sd}} g_i^{\mathrm{d}} E_i^{\mathrm{d}} \right) \text{ pooled somatic reversal potential}
\end{aligned}
$$

2 Derivation of the somatic potential distribution

We consider the prior distribution on u_{s} of the form

$$
p(u_{\mathrm{s}}|E_0, g_0) = \frac{1}{Z_0} e^{-\frac{g_0}{2\lambda_{\mathrm{e}}}(E_0 - u_{\mathrm{s}})^2} . \tag{1}
$$

We consider the dendritic likelihood functions for u_{s}:

$$
p(E_i^{\mathrm{d}}|u_{\mathrm{s}}, g_i^{\mathrm{d}}) = \frac{1}{Z_i^{\mathrm{d}}} e^{-\frac{\alpha_i^{\mathrm{sd}} g_i^{\mathrm{d}}}{2\lambda_{\mathrm{e}}}(E_i^{\mathrm{d}} - u_{\mathrm{s}})^2} . \tag{2}
$$

The posterior over u_{s} is given by

$$
p(u_{\mathrm{s}}\,|\,E_0, g_0, \boldsymbol{E}^{\mathrm{d}}, \boldsymbol{g}^{\mathrm{d}}) \;\propto\; p(\boldsymbol{E}^{\mathrm{d}}\,|\,u_{\mathrm{s}}, \boldsymbol{g}^{\mathrm{d}}) p(u_{\mathrm{s}}|E_0, g_0) = \prod_{i=1}^{D} p(E_i^{\mathrm{d}}|u_{\mathrm{s}}, g_i^{\mathrm{d}}) p(u_{\mathrm{s}}|E_0, g_0) . \tag{3}
$$

We first consider the unnormalized posterior, and rewrite it, dropping all terms constant w.r.t. u_{s}:

$$\begin{aligned}\prod_{i=1}^{D} p(E_i^{\mathrm{d}}|u_{\mathrm{s}}, g_i^{\mathrm{d}}) p(u_{\mathrm{s}}|E_0, g_0) &\propto e^{-\frac{g_0}{2\lambda_{\mathrm{e}}}(u_{\mathrm{s}}-E_0)^2} \prod_{i=1}^{D} e^{-\frac{\alpha_i^{\mathrm{sd}} g_i^{\mathrm{d}}}{2\lambda_{\mathrm{e}}}(u_{\mathrm{s}}-E_i^{\mathrm{d}})^2} \\ &\propto e^{-\frac{g_0+\sum_{i=1}^{D}\alpha_i^{\mathrm{sd}} g_i^{\mathrm{d}}}{2\lambda_{\mathrm{e}}}\left(u_{\mathrm{s}}^2 - 2u_{\mathrm{s}}\frac{g_0 E_0 + \sum_{i=1}^{D}\alpha_i^{\mathrm{sd}} g_i^{\mathrm{d}} E_i^{\mathrm{d}}}{g_0+\sum_{i=1}^{D}\alpha_i^{\mathrm{sd}} g_i^{\mathrm{d}}}\right)} \\ &\propto e^{-\frac{\bar{g}_{\mathrm{s}}}{2\lambda_{\mathrm{e}}}(u_{\mathrm{s}}-\bar{E}_{\mathrm{s}})^2} \end{aligned} \tag{4}$$

As the density needs to be normalized, we can compute the normalization factor Z directly from this form as a Gaussian integral:

$$\begin{aligned} Z &= \int du_{\mathrm{s}}\, e^{-\frac{\bar{g}_{\mathrm{s}}}{2\lambda_{\mathrm{e}}}(u_{\mathrm{s}}-\bar{E}_{\mathrm{s}})^2} \\ &= \sqrt{\frac{2\pi\lambda_{\mathrm{e}}}{\bar{g}_{\mathrm{s}}}} \end{aligned} \tag{5}$$

This finally results in the somatic potential distribution:

$$p(u_{\mathrm{s}}|\boldsymbol{W}, \boldsymbol{r}) = \frac{1}{Z} e^{-\frac{\bar{g}_{\mathrm{s}}}{2\lambda_{\mathrm{e}}}(u_{\mathrm{s}}-\bar{E}_{\mathrm{s}})^2} \,. \tag{6}$$

3 Derivation of membrane potential dynamics

We introduce the energy E as the negative logarithm of p:

$$\mathcal{E}(u_{\mathrm{s}}, \boldsymbol{W}, \boldsymbol{r}) := -\log p(u_{\mathrm{s}}|\boldsymbol{W}, \boldsymbol{r}) \,. \tag{7}$$

We obtain potential dynamics from gradient descent on $\mathcal{E}$:

$$\begin{aligned} c_{\mathrm{m}}\dot{u}_{\mathrm{s}} &= -\lambda_{\mathrm{e}}\frac{\partial}{\partial u_{\mathrm{s}}}\mathcal{E}(u_{\mathrm{s}}, \boldsymbol{W}, \boldsymbol{r}) \\ &= \lambda_{\mathrm{e}}\frac{\partial}{\partial u_{\mathrm{s}}}\log p(u_{\mathrm{s}}|\boldsymbol{W}, \boldsymbol{r}) \\ &= \lambda_{\mathrm{e}}\frac{\partial}{\partial u_{\mathrm{s}}}\left(-\frac{\bar{g}_{\mathrm{s}}}{2\lambda_{\mathrm{e}}}(u_{\mathrm{s}}-\bar{E}_{\mathrm{s}})^2 + \frac{1}{2}\log\frac{\bar{g}_{\mathrm{s}}}{2\pi\lambda_{\mathrm{e}}}\right) \\ &= \bar{g}_{\mathrm{s}}(\bar{E}_{\mathrm{s}} - u_{\mathrm{s}}) \\ &= g_0(E_0 - u_{\mathrm{s}}) + \sum_{i=1}^{D}\alpha_i^{\mathrm{sd}}\left(g_i^{\mathrm{L}}(E^{\mathrm{L}} - u_{\mathrm{s}}) + g_i^{\mathrm{E}}(E^{\mathrm{E}} - u_{\mathrm{s}}) + g_i^{\mathrm{I}}(E^{\mathrm{I}} - u_{\mathrm{s}})\right) \,. \end{aligned} \tag{8}$$

4 Derivation of weight dynamics

We want to obtain weight dynamics that approximate gradient descent on the KL:

$$-\lambda_{\mathrm{e}}\frac{\partial}{\partial W_i^{\mathrm{E/I}}}\mathbb{E}_r\left[\mathrm{KL}(p^*(u_{\mathrm{s}}|\boldsymbol{r})\|p(u_{\mathrm{s}}|\boldsymbol{W}, \boldsymbol{r}))\right] \tag{9}$$

We first rewrite the KL:

$$\begin{aligned}
\mathrm{KL}(p^*(u_\mathrm{s}|\boldsymbol{r})\|p(u_\mathrm{s}|\boldsymbol{W},\boldsymbol{r})) &= \int du_\mathrm{s}\, p^*(u_\mathrm{s}|\boldsymbol{r}) \log \frac{p^*(u_\mathrm{s}|\boldsymbol{r})}{p(u_\mathrm{s}|\boldsymbol{W},\boldsymbol{r})} \\
&= \int du_\mathrm{s}\, p^*(u_\mathrm{s}|\boldsymbol{r}) \log p^*(u_\mathrm{s}|\boldsymbol{r}) - \int du_\mathrm{s}\, p^*(u_\mathrm{s}|\boldsymbol{r}) \log p(u_\mathrm{s}|\boldsymbol{W},\boldsymbol{r}) \\
&= -S(p^*(u_\mathrm{s}|\boldsymbol{r})) - \mathbb{E}_{u_\mathrm{s}}\left[\log p(u_\mathrm{s}|\boldsymbol{W},\boldsymbol{r})\right]
\end{aligned}$$

Here, we can drop the first term as it does not depend on $\boldsymbol{W}$. We perform stochastic gradient descent in $\boldsymbol{r}$ and u_s, i.e., we drop the averages and use single samples $\boldsymbol{r} \sim p^*(\boldsymbol{r}), u^* \sim p^*(u_\mathrm{s}|\boldsymbol{r})$:

$$\begin{aligned}
\lambda_\mathrm{e} \frac{\partial}{\partial W_i^{\mathrm{E/I}}} \mathbb{E}_r\left[\mathbb{E}_{u_\mathrm{s}}\left[\log p(u_\mathrm{s}|\boldsymbol{W},\boldsymbol{r})\right]\right] =& \lambda_\mathrm{e} \frac{\partial}{\partial W_i^{\mathrm{E/I}}} \int dr\, p^*(\boldsymbol{r}) \int du_\mathrm{s}\, p^*(u_\mathrm{s}|\boldsymbol{r}) \log p(u_\mathrm{s}|\boldsymbol{W},\boldsymbol{r}) \\
\approx& \lambda_\mathrm{e} \frac{\partial}{\partial W_i^{\mathrm{E/I}}} \log p(u^*|\boldsymbol{W},\boldsymbol{r}) \ , \qquad (10)
\end{aligned}$$

where in the last step we plugged in the empirical distribution for $p^*(\boldsymbol{r})p^*(u_\mathrm{s}|\boldsymbol{r})$ consisting of Dirac-delta functions centered on the data points $(\boldsymbol{r}, u^*)$. We set

$$\dot{W}_i^{\mathrm{E/I}} = \eta \lambda_\mathrm{e} \frac{\partial}{\partial W_i^{\mathrm{E/I}}} \log p(u^*|\boldsymbol{W},\boldsymbol{r}) \qquad (11)$$

with some fixed learning rate η.

We compute the derivative:

$$\begin{aligned}
\lambda_\mathrm{e} \frac{\partial}{\partial W_i^{\mathrm{E/I}}} \log p(u_\mathrm{s}|\boldsymbol{W},\boldsymbol{r}) =& \lambda_\mathrm{e} \frac{\partial}{\partial W_i^{\mathrm{E/I}}} \left(-\frac{\bar{g}_\mathrm{s}}{2\lambda_\mathrm{e}} (u_\mathrm{s} - \bar{E}_\mathrm{s})^2 + \frac{1}{2} \log \frac{\bar{g}_\mathrm{s}}{2\pi\lambda_\mathrm{e}} \right) \\
=& -\frac{1}{2} \frac{\partial \bar{g}_\mathrm{s}}{\partial W_i^{\mathrm{E/I}}} (u_\mathrm{s} - \bar{E}_\mathrm{s})^2 - \frac{\bar{g}_\mathrm{s}}{2} \frac{\partial}{\partial W_i^{\mathrm{E/I}}} (u_\mathrm{s} - \bar{E}_\mathrm{s})^2 + \frac{\lambda_\mathrm{e}}{2} \frac{\partial}{\partial W_i^{\mathrm{E/I}}} \log \bar{g}_\mathrm{s} \qquad (12)
\end{aligned}$$

We compute the derivative:

$$\begin{aligned}
\frac{\partial \bar{g}_\mathrm{s}}{\partial W_i^{\mathrm{E/I}}} =& \frac{\partial}{\partial W_i^{\mathrm{E/I}}} \left(g_0 + \sum_{d=1}^{D} \frac{g_i^{\mathrm{sd}}}{g_i^{\mathrm{ds}} + g_i^{\mathrm{d}}} g_i^{\mathrm{d}} \right) \\
=& \frac{\partial}{\partial W_i^{\mathrm{E/I}}} \frac{g_i^{\mathrm{sd}}}{g_i^{\mathrm{ds}} + g_i^{\mathrm{d}}} g_i^{\mathrm{d}} \\
=& \left(\frac{\partial}{\partial W_i^{\mathrm{E/I}}} \frac{g_i^{\mathrm{sd}}}{g_i^{\mathrm{ds}} + g_i^{\mathrm{d}}} \right) g_i^{\mathrm{d}} + \frac{g_i^{\mathrm{sd}}}{g_i^{\mathrm{ds}} + g_i^{\mathrm{d}}} \frac{\partial}{\partial W_i^{\mathrm{E/I}}} g_i^{\mathrm{d}} \\
=& \left(-\frac{g_i^{\mathrm{sd}}}{(g_i^{\mathrm{ds}} + g_i^{\mathrm{d}})^2} \frac{\partial}{\partial W_i^{\mathrm{E/I}}} g_i^{\mathrm{d}} \right) g_i^{\mathrm{d}} + \frac{g_i^{\mathrm{sd}}}{g_i^{\mathrm{ds}} + g_i^{\mathrm{d}}} \frac{\partial}{\partial W_i^{\mathrm{E/I}}} g_i^{\mathrm{d}} \\
=& \left[\left(-\frac{g_i^{\mathrm{sd}}}{(g_i^{\mathrm{ds}} + g_i^{\mathrm{d}})^2} \right) g_i^{\mathrm{d}} + \frac{g_i^{\mathrm{sd}}}{g_i^{\mathrm{ds}} + g_i^{\mathrm{d}}} \right] r_i \\
=& \alpha_i^{\mathrm{sd}} \alpha_i^{\mathrm{ds}} r_i \qquad (13)
\end{aligned}$$

with $\alpha_i^{\mathrm{sd}} := \frac{g_i^{\mathrm{sd}}}{g_i^{\mathrm{ds}} + g_i^{\mathrm{d}}}$ and $\alpha_i^{\mathrm{ds}} := \frac{g_i^{\mathrm{ds}}}{g_i^{\mathrm{ds}} + g_i^{\mathrm{d}}}$. Note that for symmetric coupling conductances $\alpha_i^{\mathrm{sd}} = \alpha_i^{\mathrm{ds}}$.

We compute the derivative:

$$\begin{aligned}
\frac{\partial}{\partial W_i^{\mathrm{E/I}}}(u_\mathrm{s}-\bar{E}_\mathrm{s})^2 =& -2(u_\mathrm{s}-\bar{E}_\mathrm{s})\frac{\partial}{\partial W_i^{\mathrm{E/I}}}\bar{E}_\mathrm{s} \\
=& -2(u_\mathrm{s}-\bar{E}_\mathrm{s})\frac{\partial}{\partial W_i^{\mathrm{E/I}}}\left[\frac{1}{\bar{g}_\mathrm{s}}\left(g_0E_0+\sum_{d=1}^{D}\frac{g_i^\mathrm{sd}}{g_i^\mathrm{ds}+g_i^\mathrm{d}}g_i^\mathrm{d}E_i^\mathrm{d}\right)\right] \\
=& -2(u_\mathrm{s}-\bar{E}_\mathrm{s})\left(-\frac{1}{\bar{g}_\mathrm{s}}\bar{E}_\mathrm{s}\frac{\partial\bar{g}_\mathrm{s}}{\partial W_i^{\mathrm{E/I}}}+\frac{1}{g_0}\frac{\partial}{\partial W_i^{\mathrm{E/I}}}\left[\frac{g_i^\mathrm{sd}}{g_i^\mathrm{ds}+g_i^\mathrm{d}}g_i^\mathrm{d}E_i^\mathrm{d}\right]\right) \\
=& -2(u_\mathrm{s}-\bar{E}_\mathrm{s})\left(-\frac{1}{\bar{g}_\mathrm{s}}\bar{E}_\mathrm{s}\frac{\partial\bar{g}_\mathrm{s}}{\partial W_i^{\mathrm{E/I}}}+\frac{1}{\bar{g}_\mathrm{s}}\left[\frac{\partial}{\partial W_i^{\mathrm{E/I}}}\frac{g_i^\mathrm{sd}}{g_i^\mathrm{ds}+g_i^\mathrm{d}}\right]g_i^\mathrm{d}E_i^\mathrm{d}+\frac{1}{\bar{g}_\mathrm{s}}\left[\frac{g_i^\mathrm{sd}}{g_i^\mathrm{ds}+g_i^\mathrm{d}}\right]E^{\mathrm{E/I}}r_i\right) \\
=& -2(u_\mathrm{s}-\bar{E}_\mathrm{s})\left(-\frac{1}{\bar{g}_\mathrm{s}}\bar{E}_\mathrm{s}\alpha_i^\mathrm{sd}\alpha_i^\mathrm{ds}r_i-\frac{\alpha_i^\mathrm{sd}}{\bar{g}_\mathrm{s}}\left[\frac{1}{g^\mathrm{ds}+g_i^\mathrm{d}}r_i\right]g_i^\mathrm{d}E_i^\mathrm{d}+\frac{\alpha_i^\mathrm{sd}}{\bar{g}_\mathrm{s}}E^{\mathrm{E/I}}r_i\right) \\
=& -2(u_\mathrm{s}-\bar{E}_\mathrm{s})\frac{\alpha_i^\mathrm{sd}}{\bar{g}_\mathrm{s}}\left(-\bar{E}_\mathrm{s}\alpha_i^\mathrm{ds}-\left[\frac{g_i^\mathrm{d}}{g^\mathrm{ds}+g_i^\mathrm{d}}\right]E_i^\mathrm{d}+E^{\mathrm{E/I}}\right)r_i \\
=& -2(u_\mathrm{s}-\bar{E}_\mathrm{s})\frac{\alpha_i^\mathrm{sd}}{\bar{g}_\mathrm{s}}\left(E^{\mathrm{E/I}}-\left[\alpha_i^\mathrm{ds}\bar{E}_\mathrm{s}+(1-\alpha_i^\mathrm{ds})E_i^\mathrm{d}\right]\right)r_i
\end{aligned} \tag{14}$$

We compute the derivative:

$$\begin{aligned}
\frac{\partial}{\partial W_i^{\mathrm{E/I}}}\log\bar{g}_\mathrm{s} =& \frac{1}{\bar{g}_\mathrm{s}}\frac{\partial\bar{g}_\mathrm{s}}{\partial W_i^{\mathrm{E/I}}} \\
=& \frac{1}{\bar{g}_\mathrm{s}}\alpha_i^\mathrm{sd}\alpha_i^\mathrm{ds}r_i
\end{aligned} \tag{15}$$

We now put everything together, yielding:

$$\begin{aligned}
\lambda_\mathrm{e}\frac{\partial}{\partial W_i^{\mathrm{E/I}}}\log p(u^*|\boldsymbol{W},\boldsymbol{r}) =& -\frac{1}{2}\frac{\partial\bar{g}_\mathrm{s}}{\partial W_i^{\mathrm{E/I}}}(u^*-\bar{E}_\mathrm{s})^2-\frac{\bar{g}_\mathrm{s}}{2}\frac{\partial}{\partial W_i^{\mathrm{E/I}}}(u^*-\bar{E}_\mathrm{s})^2+\frac{\lambda_\mathrm{e}}{2}\frac{\partial}{\partial W_i^{\mathrm{E/I}}}\log\bar{g}_\mathrm{s} \\
=& -\frac{1}{2}\alpha_i^\mathrm{sd}\alpha_i^\mathrm{ds}r_i(u^*-\bar{E}_\mathrm{s})^2+(u^*-\bar{E}_\mathrm{s})\alpha_i^\mathrm{sd}\left(E^{\mathrm{E/I}}-\left[\alpha_i^\mathrm{ds}\bar{E}_\mathrm{s}+(1-\alpha_i^\mathrm{ds})E_i^\mathrm{d}\right]\right)r_i+\frac{1}{2}\frac{\lambda_\mathrm{e}}{\bar{g}_\mathrm{s}}\alpha_i^\mathrm{sd}\alpha_i^\mathrm{ds}r_i \\
=& \left[(u^*-\bar{E}_\mathrm{s})\left(E^{\mathrm{E/I}}-\left[\alpha_i^\mathrm{ds}\bar{E}_\mathrm{s}+(1-\alpha_i^\mathrm{ds})E_i^\mathrm{d}\right]\right)-\frac{\alpha_i^\mathrm{ds}}{2}\left((u^*-\bar{E}_\mathrm{s})^2-\frac{\lambda_\mathrm{e}}{\bar{g}_\mathrm{s}}\right)\right]\alpha_i^\mathrm{sd}r_i \\
=& \left[(u^*-\bar{E}_\mathrm{s})\left(E^{\mathrm{E/I}}-\tilde{E}_i^\mathrm{d}\right)-\frac{\alpha_i^\mathrm{ds}}{2}\left((u^*-\bar{E}_\mathrm{s})^2-\frac{\lambda_\mathrm{e}}{\bar{g}_\mathrm{s}}\right)\right]\alpha_i^\mathrm{sd}r_i
\end{aligned} \tag{16}$$

where we introduced $\tilde{E}_i^\mathrm{d}=\alpha_i^\mathrm{ds}\bar{E}_\mathrm{s}+(1-\alpha_i^\mathrm{ds})E_i^\mathrm{d}$.

5 Unreliable dendritic inputs are assigned small synaptic strengths

Here, we provide a proof that the total synaptic strength on a dendritic branch scales inversely with the presynaptic rate fluctuations. Here we explicitly consider the case of two dendritic branches.

The full loss function for two dendrites, targeted by two presynaptic rate vectors r_1 and r_2,

$$\begin{aligned}
\mathcal{L}(\boldsymbol{W}) =& \mathbb{E}_{p^*(r)}\left[\mathbb{E}_{p^*(r_1,r_2|r)}\left(\mathrm{KL}\left[p^*(u|r)||p(u|r_1,r_2,\boldsymbol{W})\right]\right)\right] \\
=& \int dr\,p^*(r)\int dr_1\,dr_2\,p^*(r_1,r_2|r)\mathrm{KL}\left[p^*(u|r)||p(u|r_1,r_2,\boldsymbol{W})\right]
\end{aligned} \tag{17}$$

can be rewritten as

$$\int dr_1\, dr_2 \int dr\, p^*(r) p^*(r_1|r) p^*(r_2|r) \int du\, p^*(u|r) \left[\log p^*(u|r) - \log p(u|r_1, r_2, \boldsymbol{W})\right] \,, \tag{18}$$

where we assumed that the input rates r_1, r_2 are conditionally independent given the ground truth rate r ($p^*(r_1, r_2|r) = p^*(r_1|r)p^*(r_2|r)$). We drop all terms which only depend on p^*, as they do not depend on the synaptic weights $\boldsymbol{W}$ on which we will perform gradients descent, thus leaving

$$-\int dr_1\, dr_2 \int dr\, p^*(r) p^*(r_1|r) p^*(r_2|r) \int du\, p^*(u|r) \log p(u|r_1, r_2) \,. \tag{19}$$

We rearrange the integrals to

$$-\int dr_1\, dr_2 \int du\, \log p(u|r_1, r_2) \int dr\, p^*(r) p^*(r_1|r) p^*(r_2|r) p^*(u|r) \,. \tag{20}$$

We now define $p^*(r), p^*(r_i|r)$: the distribution over ground truth rates r is a Gaussian with arbitrary mean and variance, the distribution over input rates r_i are Gaussians around the ground truth r with "modality-specific" variances σ_i^2

$$p^*(r) := \frac{1}{\sqrt{2\pi\sigma_r^2}} e^{-\frac{1}{2\sigma_r^2}(r-\mu_r)^2} \,, \tag{21}$$

$$p^*(r_i|r) := \frac{1}{\sqrt{2\pi\sigma_i^2}} e^{-\frac{1}{2\sigma_i^2}(r_i-r)^2} \,. \tag{22}$$

We can rewrite the product of Gaussians appearing in the loss function in the last integral over r (see also [1])

$$\begin{aligned} p^*(r)p^*(r_1|r)p^*(r_2|r) =& \frac{1}{\sqrt{2\pi\sigma_r^2}} e^{-\frac{1}{2\sigma_r^2}(r-\mu_r)^2} \frac{1}{\sqrt{2\pi\sigma_1^2}} e^{-\frac{1}{2\sigma_1^2}(r_1-r)^2} \frac{1}{\sqrt{2\pi\sigma_2^2}} e^{-\frac{1}{2\sigma_2^2}(r_2-r)^2} \\ =& C(\mu_r, \sigma_r, r_1, \sigma_1, r_2, \sigma_2) \frac{1}{\sqrt{2\pi\sigma^2}} e^{-\frac{1}{2\sigma^2}(r-\mu)^2} \end{aligned} \tag{23}$$

with

$$C(\mu_r, \sigma_r, r_1, \sigma_1, r_2, \sigma_2) := \frac{\sqrt{2\pi\sigma^2}}{\sqrt{2\pi\sigma_r^2}\sqrt{2\pi\sigma_1^2}\sqrt{2\pi\sigma_2^2}} e^{\frac{1}{2}\sigma^2\left(\frac{r_1}{\sigma_1^2}+\frac{r_2}{\sigma_2^2}\right)^2 - \frac{1}{2}\left(\frac{r_1^2}{\sigma_1^2}+\frac{r_2^2}{\sigma_2^2}\right)} \,, \tag{24}$$

$$\frac{1}{\sigma^2} := \frac{1}{\sigma_r^2} + \frac{1}{\sigma_1^2} + \frac{1}{\sigma_2^2} \,, \tag{25}$$

$$\mu := \sigma^2 \left(\frac{\mu_r}{\sigma_r^2} + \frac{r_1}{\sigma_1^2} + \frac{r_2}{\sigma_2^2} \right) \,. \tag{26}$$

For simplicity we consider a target distribution of the somatic voltage given the ground truth rate r that is delta function

$$p^*(u|r) := \delta(u - r) \,. \tag{27}$$

With this definition we can solve the integral over r in the loss function

$$\int dr\, p^*(r) p^*(r_1|r) p^*(r_2|r) p^*(u|r) = C(\mu_r, \sigma_r, r_1, \sigma_1, r_2, \sigma_2) \frac{1}{\sqrt{2\pi\sigma^2}} e^{-\frac{1}{2\sigma^2}(u-\mu)^2} , \tag{28}$$

and our loss function thus becomes

$$-\int dr_1\, dr_2\, C(\mu_r, \sigma_r, r_1, \sigma_1, r_2, \sigma_2) \int du\, \underbrace{\frac{1}{\sqrt{2\pi\sigma^2}} e^{-\frac{1}{2\sigma^2}(u-\mu)^2}}_{p^*(u|r_1,r_2)} \log p(u|r_1, r_2) . \tag{29}$$

Since for learning we will consider derivatives w.r.t. synaptic weights $\boldsymbol{W}$, we can add a term independent of synaptic weights ($\int du\, p^*(u|r_1,r_2) \log p^*(u|r_1,r_2)$) to again obtain an objective function involving a KL

$$-\int dr_1\, dr_2\, C(\mu_r, \sigma_r, r_1, \sigma_1, r_2, \sigma_2) \mathrm{KL}\left[p^*(u|r_1,r_2) || p(u|r_1,r_2)\right] . \tag{30}$$

We want to compare the relative influence of the input noise amplitudes σ_i^2 on the target distribution with the influence of synaptic weights $\boldsymbol{W}$ on the distribution represented by the neuron. To achieve this, we consider a Taylor expansion of both $p^*(u|r_1,r_2)$ and $p(u|r_1,r_2)$ around the input rates up to second order and compare coefficients of this expansion. Synaptic plasticity in our model tries to match these two distribution, hence we assume that it also matches these coefficients by minimizing their KL. For simplicity, we assume $\lambda_\mathrm{e} = 1$ in the following.

We compute the first derivative of $p^*(u|r_1,r_2)$ which under our assumptions takes Gaussian form w.r.t. r_1

$$\begin{aligned}
\frac{\partial}{\partial r_1} p^*(u|r_1,r_2) &= p^*(\cdot|\cdot) \frac{\partial}{\partial r_1} \left(-\frac{1}{2\sigma^2}(u-\mu)^2 \right) \\
&= p^*(\cdot|\cdot) \left(\frac{1}{\sigma^2}(u-\mu)\sigma^2 \frac{1}{\sigma_1^2} \right) \\
&= p^*(\cdot|\cdot) \left(\frac{1}{\sigma_1^2}(u-\mu) \right) .
\end{aligned} \tag{31}$$

Next we compute the second derivative

$$\begin{aligned}
\frac{\partial^2}{\partial r_1^2} p^*(u|r_1,r_2) &= \frac{\partial}{\partial r_1} \left(p^*(\cdot|\cdot) \left(\frac{1}{\sigma_1^2}(u-\mu) \right) \right) \\
&= \left(\frac{\partial}{\partial r_1} p^*(\cdot|\cdot) \right) \frac{1}{\sigma_1^2}(u-\mu) + p^*(\cdot|\cdot) \frac{\partial}{\partial r_1} \left(\frac{1}{\sigma_1^2}(u-\mu) \right) \\
&= p^*(\cdot|\cdot) \left(\frac{1}{\sigma_1^2}(u-\mu) \right)^2 - p^*(\cdot|\cdot) \frac{\sigma^2}{\sigma_1^4} .
\end{aligned} \tag{32}$$

Similarly, we compute the first derivative of $p(u|r_1,r_2)$ (see Eqn. **??**)

$$\begin{aligned}
\frac{\partial}{\partial r_1} p(u|r_1,r_2) &= \frac{1}{\sqrt{2\pi}} \left(\frac{\partial}{\partial r_1} \sqrt{g} \right) e^{\cdot} + p(\cdot|\cdot) \frac{\partial}{\partial r_1} \left(-\frac{g}{2}(u-\mu)^2 \right) \\
&= \frac{1}{\sqrt{2\pi}} \frac{1}{2\sqrt{g}} (w_1^\mathrm{E} + w_1^\mathrm{I}) e^{\cdot} + p(\cdot|\cdot) \left(-\frac{1}{2}(w_1^\mathrm{E} + w_1^\mathrm{I})(u-\mu)^2 + g(u-\mu)\frac{\partial}{\partial r_1}\mu \right) \\
&= p(\cdot|\cdot) \left(\frac{1}{2g}(w_1^\mathrm{E} + w_1^\mathrm{I}) - \frac{1}{2}(u-\mu)^2 (w_1^\mathrm{E} + w_1^\mathrm{I}) + (u-\mu)(w_1^\mathrm{E} E^\mathrm{E} + w_1^\mathrm{I} E^\mathrm{I} - \mu(w_1^\mathrm{E} + w_1^\mathrm{I})) \right) \\
&= p(\cdot|\cdot) \left(\frac{1}{2} \left(\frac{1}{g} - (u-\mu)^2 \right) (w_1^\mathrm{E} + w_1^\mathrm{I}) + (u-\mu)(w_1^\mathrm{E} E^\mathrm{E} + w_1^\mathrm{I} E^\mathrm{I} - \mu(w_1^\mathrm{E} + w_1^\mathrm{I})) \right) .
\end{aligned} \tag{33}$$

Taking the second derivative yields

$$
\begin{aligned}
\frac{\partial^2}{\partial r_1^2} p(u|r_1,r_2) =& \left(\frac{\partial}{\partial r_1} p(\cdot|\cdot)\right)(\cdot) + p(\cdot|\cdot)\left(\frac{\partial}{\partial r_1}(\cdot)\right) \\
=& p(\cdot|\cdot)\,(\cdot)^2 + p(\cdot|\cdot)\Bigg(\frac{1}{2}\left(\left(\frac{\partial}{\partial r_1}\frac{1}{g}\right) - \left(\frac{\partial}{\partial r_1}(u-\mu)^2\right)\right)(w_1^{\mathrm{E}} + w_1^{\mathrm{I}}) \\
&+ \left(\frac{\partial}{\partial r_1}(u-\mu)\right)(w_1^{\mathrm{E}}E^{\mathrm{E}} + w_1^{\mathrm{I}}E^{\mathrm{I}} - \mu(w_1^{\mathrm{E}} + w_1^{\mathrm{I}})) \\
&- (u-\mu)\left(\frac{\partial}{\partial r_1}\mu\right)(w_1^{\mathrm{E}} + w_1^{\mathrm{I}})\Bigg) \\
=& p(\cdot|\cdot)\,(\cdot)^2 + p(\cdot|\cdot)\Bigg(\frac{1}{2}\Bigg(-\frac{1}{g^2}(w_1^{\mathrm{E}} + w_1^{\mathrm{I}}) \\
&+ 2(u-\mu)\frac{1}{g}(w_1^{\mathrm{E}}E^{\mathrm{E}} + w_1^{\mathrm{I}}E^{\mathrm{I}} - \mu(w_1^{\mathrm{E}} + w_1^{\mathrm{I}}))\Bigg)(w_1^{\mathrm{E}} + w_1^{\mathrm{I}}) \\
&- \left(\frac{1}{g}(w_1^{\mathrm{E}}E^{\mathrm{E}} + w_1^{\mathrm{I}}E^{\mathrm{I}} - \mu(w_1^{\mathrm{E}} + w_1^{\mathrm{I}}))\right)(w_1^{\mathrm{E}}E^{\mathrm{E}} + w_1^{\mathrm{I}}E^{\mathrm{I}} - \mu(w_1^{\mathrm{E}} + w_1^{\mathrm{I}})) \\
&- (u-\mu)\frac{1}{g}(w_1^{\mathrm{E}}E^{\mathrm{E}} + w_1^{\mathrm{I}}E^{\mathrm{I}} - \mu(w_1^{\mathrm{E}} + w_1^{\mathrm{I}}))(w_1^{\mathrm{E}} + w_1^{\mathrm{I}})\Bigg) \\
=& p(\cdot|\cdot)\,(\cdot)^2 + p(\cdot|\cdot)\Bigg(-\frac{1}{2g^2}(w_1^{\mathrm{E}} + w_1^{\mathrm{I}})^2 \\
&+ (u-\mu)\frac{1}{g}(w_1^{\mathrm{E}}E^{\mathrm{E}} + w_1^{\mathrm{I}}E^{\mathrm{I}} - \mu(w_1^{\mathrm{E}} + w_1^{\mathrm{I}}))(w_1^{\mathrm{E}} + w_1^{\mathrm{I}}) \\
&- \frac{1}{g}(w_1^{\mathrm{E}}E^{\mathrm{E}} + w_1^{\mathrm{I}}E^{\mathrm{I}} - \mu(w_1^{\mathrm{E}} + w_1^{\mathrm{I}}))^2 \\
&- (u-\mu)\frac{1}{g}(w_1^{\mathrm{E}}E^{\mathrm{E}} + w_1^{\mathrm{I}}E^{\mathrm{I}} - \mu(w_1^{\mathrm{E}} + w_1^{\mathrm{I}}))(w_1^{\mathrm{E}} + w_1^{\mathrm{I}})\Bigg) \\
=& p(\cdot|\cdot)\,(\cdot)^2 + p(\cdot|\cdot)\left(-\frac{1}{2g^2}(w_1^{\mathrm{E}} + w_1^{\mathrm{I}})^2 - \frac{1}{g}(w_1^{\mathrm{E}}E^{\mathrm{E}} + w_1^{\mathrm{I}}E^{\mathrm{I}} - \mu(w_1^{\mathrm{E}} + w_1^{\mathrm{I}}))^2\right). \qquad (34)
\end{aligned}
$$

Now we compare coefficients of the Taylor expansions in r_1 around r_2, i.e., we assume that input noise amplitudes are small. From the zeroth order we obtain

$$
p^*(u|r_1,r_2)\big|_{r_1=r_2} = p(u|r_1,r_2)\big|_{r_1=r_2}\,. \qquad (35)
$$

From the first order (Eqs.31 & 33) we obtain

$$
\begin{aligned}
p^*(u|r_1,r_2)\frac{1}{\sigma_1^2}(u-\mu)\bigg|_{r_1=r_2} &= p(u|r_1,r_2)\left(\frac{1}{2}\left(\frac{1}{g} - (u-\mu)^2\right) + (u-\mu)(E_1-\mu)\right)(w_1^{\mathrm{E}} + w_1^{\mathrm{I}})\bigg|_{r_1=r_2} \\
\frac{1}{\sigma_1^2}(u-\mu)\bigg|_{r_1=r_2} &= \left(\frac{1}{2}\left(\frac{1}{g} - (u-\mu)^2\right) + (u-\mu)(E_1-\mu)\right)(w_1^{\mathrm{E}} + w_1^{\mathrm{I}})\bigg|_{r_1=r_2}\,. \qquad (36)
\end{aligned}
$$

where we used the result from the zeroth order to cancel $p^*(u|r_1,r_2)$ with $p(u|r_1,r_2)$ and introduced

$E_1 := \frac{w_1^{\mathrm{E}} E^{\mathrm{E}} + w_1^{\mathrm{I}} E^{\mathrm{I}}}{w_1^{\mathrm{E}} + w_1^{\mathrm{I}}}$. Finally, from the second order (Eqs.32 & 34) we obtain

$$p^*(\cdot|\cdot)\left(\frac{1}{\sigma_1^2}(u-\mu)\right)^2 - p^*(\cdot|\cdot)\frac{\sigma^2}{\sigma_1^4}\bigg|_{r_1=r_2} = p(\cdot|\cdot)(\cdot)^2 + p(\cdot|\cdot)\left(-\frac{1}{2g^2} - \frac{1}{g}(E_1-\mu)^2\right)(w_1^{\mathrm{E}} + w_1^{\mathrm{I}})^2\bigg|_{r_1=r_2}$$

$$\frac{\sigma^2}{\sigma_1^4} = \left(\frac{1}{2g^2} + \frac{1}{g}(E_1-\mu)^2\right)(w_1^{\mathrm{E}} + w_1^{\mathrm{I}})^2\bigg|_{r_1=r_2}. \quad (37)$$

Similarly, we consider an expansion in r_2 around r_1 to obtain an expression similar to the previous line. We divide these two equations to obtain

$$\frac{\sigma_2^4}{\sigma_1^4} = \frac{\left(\frac{1}{2g^2} + \frac{1}{g}(E_1-\mu)^2\right)w_1^2\Big|_{r_1=r_2}}{\left(\frac{1}{2g^2} + \frac{1}{g}(E_2-\mu)^2\right)w_2^2\Big|_{r_1=r_2}}. \quad (38)$$

Both dendrites are learning to match the same target potentials, hence, we assume that the ratio of excitation and inhibition is identical for both dendrites and thus $E_1 = E_2$. This corresponds to the general setting where the inputs to the both dendrites are not perfectly correlated, and each dendrite thus learns to match the target potential. With this, the equation simplifies to (after taking the square root)

$$\frac{\sigma_2^2}{\sigma_1^2} = \frac{w_1}{w_2}. \quad (39)$$

We thus conclude that synaptic plasticity, i.e., stochastic gradient descent on our loss function, not only allows the neuron to match the target distribution, but that in this process it also aligns synaptic weights such that more reliable inputs receive larger synaptic weights.

6 Dendritic parameters

Our approach relies on two assumptions with respect to the biophysical model (Eqs. **??**, **??**): the capacitances of the dendritic compartments are small compared to the somatic capacitance and the dendritic conductances g_i^{d} are able to overrule the somatic prior g_0. A recently developed dendritic simplification framework [2] allows us to systematically reduce full biophysical models to obtain the parameters of the reduced compartmental models (Eqs. **??**, **??**) used in this work. Given a set of dendritic locations on the morphology along the dendritic tree, this approach yields capacitances, leak conductances and coupling conductances for the simplified model that optimally reproduce the dynamics of the full model, at those chosen locations (Fig. 1a). This, in turns, allows us to assert the validity of the aforementioned assumptions.

We use a detailed biophysical model of an L5 pyramidal cell [3]. Without synaptic input, the ion channels in this model collectively determine the cell's prior, encoded in the resting membrane potential and the total conductance at rest. Per dendritic segment, we aggregate these conductance contributions into a single, prior conductance. Formally, this conductance is a passive leak, and the resulting model is a passive model with the same prior (and morphology) as the detailed model.

Then, we choose dendritic sites that allow us to test the validity of our assumptions. The morphology has seven basal dendritic subtrees with branches of at least 200μm. In each subtree, we select one such branch (green in Fig. 1a), and place a single dendritic location on each of those branches at a given distance

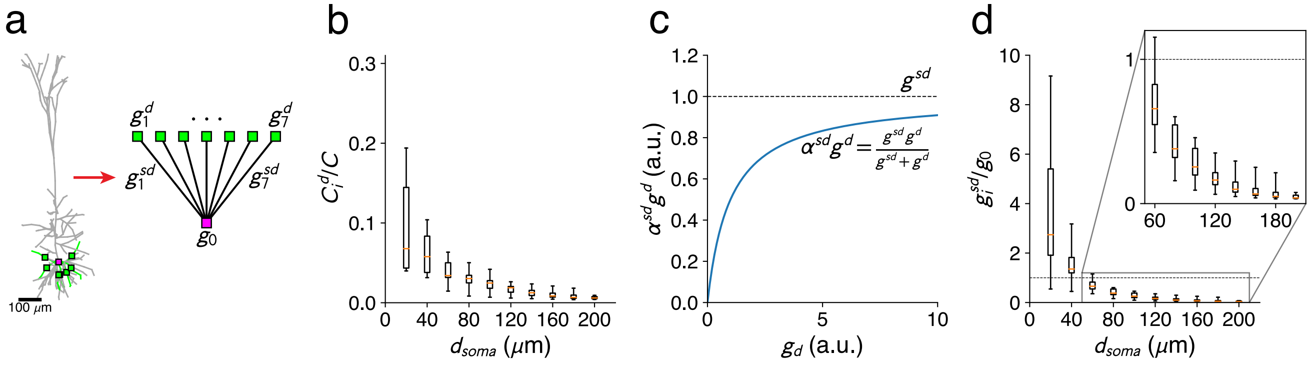

Figure 1: **Parameters of the reduced compartmental model as derived from a detailed morphological model.** **(a)** A detailed L5 Pyramidal cell model (left) is reduced to a configuration with one dendritic compartment on each of seven main basal subtrees (right). **(b)** Ratio of dendritic to somatic capacitance, for increasing distances between the dendritic sites and the soma. The box indicates the lower and upper quartile values and the orange bar the median. The whiskers indicate the minimal and maximal values. The ratio is always much smaller than one, supporting our approximation of using the instantaneous solution for the dendritic voltage. **(c)** Effective dendritic conductance at the soma, $\alpha_i^{sd} g_i^d$, as a function of the isolated dendritic conductance g_i^d. This quantity represents the effective reliability of the dendritic potential as read out at the soma. It saturates at the level of the somato-dendritic coupling conductance g_i^{sd}. **(d)** Ratio of the somato-dendritic coupling conductance to the somatic leak conductance for increasing distance between the dendritic site and the soma. When this ratio is larger than one, a single branch can overrule the somatic prior. Otherwise, multiple branches have to cooperate to overrule the prior. The inset shows a magnified version for dendritic sites farther than 50 μm from the soma.

from the soma. We increase the distance between soma and dendritic sites in increments of 20 μm and derive a reduced compartmental model for each configuration (Fig. 1a). We then compare the ratios of dendritic capacitance C_i^d and somatic capacitance C for the seven compartments $i \in \{1, \ldots, 7\}$. We find that these ratio are much smaller than one, no matter the distance from the soma (Fig. 1b).

Then, we asses the theoretical maximum degree to which synapses placed at the dendritic sites under investigation can contribute to overruling the somatic prior. The effective dendritic conductance of compartment i, measured at the soma, is given by $\alpha_i^{sd} g_i^d$ (Eqn. **??**). This function has an asymptotic maximum at the dendro-somatic coupling conductance g_i^{sd} (Fig. 1c). In consequence, g_i^{sd} is the theoretical maximal conductance that dendritic synapses in compartment i can exert at the soma. We thus need to compare g_i^{sd} with the somatic prior g_0 (Fig. 1d). For a distance between soma and dendritic site smaller than ~ 50μm, we find that a single branch can overrule the prior, as the ratio g_i^{sd}/g_0 is typically larger than one. For larger distances, multiple branches have to collaborate to overrule the prior (Fig. 1D, inset).

References

1. Bromiley, P. Products and Convolutions of Gaussian Probability Density Functions, Tina. *Memo* (2018).

2. Wybo, W. A. *et al.* Data-driven reduction of dendritic morphologies with preserved dendro-somatic responses. *eLife* **10,** 1–26 (2021).

3. Hay, E., Hill, S., Schrmann, F., Markram, H. & Segev, I. Models of neocortical layer 5b pyramidal cells capturing a wide range of dendritic and perisomatic active properties. *PLoS Computational Biology* **7,** e1002107 (July 2011).
