# Supplementary material for: Conductance-based dendrites perform Bayes-optimal cue integration
Source: PLoS Comput Biol. 2024 Jun 12;20(6):e1012047. doi: 10.1371/journal.pcbi.1012047 (PMC11168673; doi:10.1371/journal.pcbi.1012047)
Supplement: Attachment — Submitted filename: Review_PLOS.pdf [file pcbi.1012047.s002.pdf]

Review for "Learning Bayes-optimal dendritic opinion pooling"

Overall

Research Summary: The authors begin this paper with the focus that single neurons fulfill computations, and their problem statement is that the computational role of the biophysical property of dynamic membrane conductances remains unclear. This paper does well to elevate the significance of membrane conductances by demonstrating that they could play a role in their proposed analogy that **single neurons with dendrites perform Bayesian opinion weighting**. The authors make a correspondence between biophysical aspects of a single neuron (such as local dendritic membrane potential, local synaptic conductance, and somatic output) and terms involved in the computation of Bayesian opinion weighting (opinion, reliability, and decisions respectively). The authors derive a plasticity rule based on this framework that employs stochastic gradient descent, and go further to rederive biophysical dynamics from the normative direction using the Bayesian opinion weighting framework. The authors continue by performing several simulations that reproduce behavioral data and end by making several testable predictions.

Strengths: This paper is a thorough proposal of this analogy, and takes the time to clearly build the theory for single neuron computation that includes dendrites and synapses. In the introduction, the authors summarize their findings and some key literature establishing the context for their contribution. In the results, the authors do well to organize and build their proposed theory, and illustrate corresponding conclusions from simulations and their reasoning both in text and through well-composed and clear figures. In the discussion, the authors give further review of the literature with the purpose of expanding the proposed theory's applicability to other dendritic computations. The authors also consider further conclusions based on their theory, including the role of NMDA and calcium spikes, and testable experimental predictions. In particular, the authors consider an alternative to their proposed plasticity rule and explain how their proposed role is more generalizable. The methods are also polished and incredibly clear.

Weaknesses: Before listing the weaknesses of the paper, **I state that they are not major weaknesses**, and that all critiques are toward the aim of strengthening the paper through improving clarity and adding reinforcing justifications.

Of the weaknesses I did see, there was a reoccuring need for justifying certain assumptions. The most significant in my view was the assumption that one entire dendritic subtree (a subtree defined as a dendritic branch connected to the soma) corresponds to exactly one compartment (and as such, one opinion and one reliability). Although this assumption adds simplicity and power to the proposed theory, this is a key assumption that should be justified given the many biophysical phenomena that occur due to synaptic interactions and non-interactions within a single dendritic subtree. I recommend the authors to either provide a justification for this simplification or provide discussion of this as a point of departure for future research to accommodate biological complexity. More thoughts and details on this are in the discussion of areas for improvements (**see point 2.2.2**).

Other more minor assumptions caught my attention and are opportunities for the authors to address with justifications, to explain the limitations of the theory, and to point the way forward for further development of this theory. I noticed the discussion section could be improved with a treatment of the limitations of the framework, particularly that which comes from tension through simplifying biological complexity.

Recommended action: I recommend acceptance with minor revisions

Discussion of specific areas for improvement → *All of the following areas for improvement are minor*.

1. Introduction:
    - 1.1. **Literature: Alternative theories** → Page 2, para 2: The authors use this section to introduce the context and contributions of their work. When the authors write that "the biophysics of the bidirectional current flow in cortical neurons with multiple dendritic compartments naturally implements Bayesian opinion weighting", I was left wondering if there were any other alternative analogies in the literature. I suggest the authors address if there are any other alternative metaphors or analogies for single neuron function, particularly with dendrites, and justify why their proposed analogy of Bayesian opinion weighting may have particular advantages over others. This could be addressed in either the introduction or the discussion.
    - 1.2. **Assumption/Justification: Recurrence** → Page 2, para 3: The authors state, "While the dendritic opinion weighting emerges from the recurrent interaction of multiple compartments within the dendritic tree, at the level of the input-output transfer function, the effective computation can be described in a feed-forward manner". Here, and and in the results section, the authors treat the computation of the neuron with dendrites as feedforward, despite its recurrency.
    On Page 9, para 1: "We hypothesize that the backpropagating action potential rate that codes for u_s^* can influence dendritic synapses." Spatial properties through which the bAP impacts the dendritic tree, would need to be addressed with some recurrent interaction of multiple compartments. I recommend a discussion of recurrence as a limitation of the proposed framework and a need to address recurrence in future models that may explain bAP's role in setting the target input.
2. Results:
    - 2.1. Opinion weighting in cortical neurons
        - 2.1.1. **Clarity** → Page 3, para 1: "In the absence of stimuli, the 'cat neuron' has a low prior opinion…". As a reader I was confused if the "cat neuron" corresponded to the bimodal (VA, see fig 2) input neuron. I suggest making this a little clearer.
        - 2.1.2. **Clarity** → Fig. 2: Is the neuron in 2d and 2e different from the neuron 2c? If they are different, is the neuron in 2d/2e receiving input from the neuron 2c as the VA input? In addition, is the VA input the "cat neuron" or is the neuron in 2d/2e the "cat neuron"? I suggest making this a little clearer.
        - 2.1.3. **Clarity** → Fig. 2e: Is the opinion (which is labeled the "somatic membrane potential of the cat neuron" in page 3, para 1) interchangeable between E^d and u^d? I suggest making this a little clearer
    - 2.2. The neuronal opinion code
        - 2.2.1. **Limitations/Scope: Non-monotonic transfer functions** → Page 6, para 2: "In our framework the somatic membrane potential is transformed into an instantaneous output rate… via some monotonically increasing transfer function…" It is fair to say in this particular framework this transfer function can be monotonically increasing. However, it is known that non-monotonic dendritic phenomena, such as dCaAPs (increasing excitatory input intensity can yield decreasing somatic depolarization) (Gidon et al., 2020), can result in very interesting dynamics that would fall outside of this framework. I recommend some treatment of these non-monotonic response phenomena in the discussion when addressing the list of phenomena that do fall within this framework. As a reviewer, I understand thorough treatment of this subject may fall outside of the scope of this work, however acknowledging these phenomena may be a good communication of the potential limitations of this analogy.Stimuli elicit neuron-specific opinions and increase the neuronal reliability

2.2.2. **Assumption/Justification: Subtree Simplification** → Page 6, para 4: This paragraph describes the opinion weighting calculation within the neuron with dendrites. In it, it assumes that there is one opinion per dendritic subtree. This assumes there cannot be 2 opinions on a single subtree, which removes the need to consider spatial effects on the interaction of 2 or more opinions within one subtree.

2.2.2.1. I would like to see the justification for this simplifying assumption, given the phenomena of superlinearity (Polsky et al., 2004) from synaptic interactions within dendritic subtrees and synaptic clustering (Kastellakis & Poirazi, 2019) in general that may be important for the computations within the single neuron. Otherwise, I would like to see in the discussion a treatment of this simplifying discussion, as well as how it may or may not be a point of departure for future research and development of the proposed theory.

2.2.2.2. Page 8, para 2: In the section "Bayesian neural dynamics", the authors write that the models are "bidirectionally-coupled multi-compartment models with leaky integrator dynamics and conductance-based synaptic coupling under the assumption of fast dendritic responses." Here, it shows why inter-synaptic interactions are not considered in this framework's development.

2.2.2.3. Page 14, Eq 6: In the Methods, in the section "Equivalent somato-dendritic circuit", the authors give an equation for the dynamics of the somatic potential u_s, which is a linear sum of the soma compartment conductance and the dendritic conductances. The authors use this as a premise for the rest of their derivation for their framework. Treating entire dendritic subtrees as singular compartments simplifies away spatiotemporal relationships between synapses. It is fair to make simplifications like this, however as a reader I am looking for a justification for this abstraction, given the complexity of biological reality. I recommend treatment of this assumption in the discussion.

2.2.3. **Clarity** →Page 7, para 2: "increasing stimulus reliability". As a reader, I was confused by the language of "reliability" here given that reliability of opinion is invoked earlier. I would like to see how the 'reliability of opinion' and 'reliability of the stimulus' relate, if at all. **clarity**

2.3. Learning Bayes-optimal cue combinations

2.3.1. **Expansion: Cross-validation and Potential Overfitting** → Fig. 7: The caption of this figure, states that each experiment consisted of 20,000 trials. After looking through the supporting information, I do not see figures showing how learning performance improved over time through loss or training curves, often used in machine learning literature. This raises my concern that the network model may be overfit to the target, which could increase the "average loss in %" in figure 7c derived from the test trials. Though not necessarily important for the figure in the main text of the paper, I would like to see data checking for potential overfitting in the supplementary information. If there is overfitting, perhaps the authors could consider an early-stopping policy using cross-validation when training the network model. The suggestions may result in determining if the bimodal cues, the visual cues only, or the tactile cues only could yield more efficient learning of the target.

3. Discussion

3.1. **Claim/Scope: Dendritic Computations** → Page 12, para 3: "All of these computations could be put into our framework of opinion pooling, where the calculations on different dendrites are combined according to their reliabilities." As a reader, I'm wondering if the authors are leaving these computations as exercises for the reader. I am not sure if making this claim without necessarily spelling them out elsewhere in the paper is sufficient for the discussion section of

this paper; however, I would be satisfied if it were made clear that demonstrating these computations is outside of the scope of the paper.

3.2. **Claim/Scope: Single Neurons to Populations** → Page 13, para 3: "In our framework, to read out the reliability of a single neuron's opinion about the presence of its presynaptic feature, postsynaptic neurons either have to average across time or across a population of neurons that encode the same feature. *Our single-neuron description of Bayesian inference is complementary to these population-based models*." I am not sure if making this claim without necessarily spelling it out elsewhere in the paper is sufficient for the discussion section of this paper; however I would be satisfied if it were made clear that demonstrating this complementarity is outside of the scope of the paper.

3.3. In general, the authors could have given more attention to assumptions and limitations of their framework and used this point of discussion to suggest future work.

References

Gidon, A., Zolnik, T. A., Fidzinski, P., Bolduan, F., Papoutsi, A., Poirazi, P., Holtkamp, M., Vida, I., & Larkum, M. E. (2020). Dendritic action potentials and computation in human layer 2/3 cortical neurons. *Science*, *367*(6473), 83–87. https://doi.org/10.1126/science.aax6239

Kastellakis, G., & Poirazi, P. (2019). Synaptic Clustering and Memory Formation. *Frontiers in Molecular Neuroscience*, *12*. https://www.frontiersin.org/articles/10.3389/fnmol.2019.00300

Polsky, A., Mel, B. W., & Schiller, J. (2004). Computational subunits in thin dendrites of pyramidal cells. *Nature Neuroscience*, *7*(6), 621–627. https://doi.org/10.1038/nn1253
