# Supplementary material for: Conductance-based dendrites perform Bayes-optimal cue integration
Source: PLoS Comput Biol. 2024 Jun 12;20(6):e1012047. doi: 10.1371/journal.pcbi.1012047 (PMC11168673; doi:10.1371/journal.pcbi.1012047)
Supplement: Attachment — Submitted filename: reply_letter.pdf [file pcbi.1012047.s003.pdf]

Jakob Jordan
Department of Physiology
University of Bern
Bühlplatz 5, CH-3012 Bern
Switzerland
email: jakob.jordan@unibe.ch

September 20, 2023

Blake A Richards
Academic Editor, PLOS Computational Biology
Thomas Serre
Section Editor, PLOS Computational Biology

Resubmission of manuscript PCOMPBIOL-D-22-01785

Dear Blake A Richards, dear Thomas Serre,

please find enclosed our revised manuscript *"Conductance-based dendrites perform Bayes-optimal cue integration"*, which we resubmit for publication in PLOS Computational Biology. We would like to thank you and the reviewers for valuable feedback. We believe this helped us significantly improve the quality of the manuscript.

The reviewer's main concern was the accessibility of the manuscript. We have thus significantly revised both the manuscript's structure as well as the description of our approach. Accordingly we have rewritten large parts of the introduction, results, and the discussion. We hope that the revised manuscript is more accessible to a wider audience.

All concerns of the reviewers have been addressed and the manuscript was adapted accordingly. A point-by-point reply for each reviewer is provided below.

Best regards,
Jakob Jordan (on behalf of all authors)

encl: - reply to reviewer #1
- reply to reviewer #2
- revised manuscript

Reply to comments of reviewer #1

(1) Research Summary: *The authors begin this paper with the focus that single neurons fulfill computations, and their problem statement is that the computational role of the biophysical property of dynamic membrane conductances remains unclear. This paper does well to elevate the significance of membrane conductances by demonstrating that they could play a role in their proposed analogy that single neurons with dendrites perform Bayesian opinion weighting. The authors make a correspondence between biophysical aspects of a single neuron (such as local dendritic membrane potential, local synaptic conductance, and somatic output) and terms involved in the computation of Bayesian opinion weighting (opinion, reliability, and decisions respectively). The authors derive a plasticity rule based on this framework that employs stochastic gradient descent, and go further to rederive biophysical dynamics from the normative direction using the Bayesian opinion weighting framework. The authors continue by performing several simulations that reproduce behavioral data and end by making several testable predictions.*

We thank the reviewer for their comments and constructive critique. Below we have addressed all issues raised by the reviewer in a point-to-point reply.

(2) Strengths: *This paper is a thorough proposal of this analogy, and takes the time to clearly build the theory for single neuron computation that includes dendrites and synapses. In the introduction, the authors summarize their findings and some key literature establishing the context for their contribution. In the results, the authors do well to organize and build their proposed theory, and illustrate corresponding conclusions from simulations and their reasoning both in text and through well-composed and clear figures. In the discussion, the authors give further review of the literature with the purpose of expanding the proposed theory's applicability to other dendritic computations. The authors also consider further conclusions based on their theory, including the role of NMDA and calcium spikes, and testable experimental predictions. In particular, the authors consider an alternative to their proposed plasticity rule and explain how their proposed role is more generalizable. The methods are also polished and incredibly clear.*

We thank the reviewer for this positive feedback.

(3) Weaknesses: *Before listing the weaknesses of the paper, I state that they are not major weaknesses, and that all critiques are toward the aim of strengthening the paper through improving clarity and adding reinforcing justifications. Of the weaknesses I did see, there was a reoccuring need for justifying certain assumptions. The most significant in my view was the assumption that one entire dendritic subtree (a subtree defined as a dendritic branch connected to the soma) corresponds to exactly one compartment (and as such, one opinion and one reliability). Although this assumption adds simplicity and power to the proposed theory, this is a key assumption that should be justified given the many biophysical phenomena that occur due to synaptic interactions and non-interactions within a single dendritic subtree. I recommend the authors to either provide a justification for this simplification or provide discussion of this as a point of departure for future research to accommodate biological complexity. More thoughts and details on this are in the discussion of areas for improvements (see point 2.2.2). Other more minor assumptions caught my attention and are opportunities for the authors to address with justifications, to explain the limitations of the theory, and to point the way forward for further development of this theory. I noticed the discussion section could be improved with a treatment of the limitations of the framework, particularly that which comes from tension through simplifying biological complexity.*

We appreciate this constructive criticism and provide detailed replies below.

(4) Recommended action: *I recommend acceptance with minor revisions*

We thank the reviewer for this positive evaluation.

(5) *Discussion of specific areas for improvement → All of the following areas for improvement are minor.*

1. Introduction:

1.1. **Literature***: Alternative theories → Page 2, para 2: The authors use this section to introduce the context and contributions of their work. When the authors write that "the biophysics of the bidirectional current flow in cortical neurons with multiple dendritic compartments naturally implements Bayesian opinion weighting", I was left wondering if there were any other alternative analogies in the literature. I suggest the authors address if there are any other alternative metaphors or analogies for single neuron function, particularly with dendrites, and justify why their proposed analogy of Bayesian opinion weighting may have particular advantages over others. This could be addressed in either the introduction or the discussion.*

Indeed, several other functions for dendrites have been proposed in the literature. In the discussion we now include a brief discussion of the integration of our theory with theories that assign an important role to dendrites in the context of learning.

(6) *1.2.* **Assumption/Justification***: Recurrence → Page 2, para 3: The authors state, "While the dendritic opinion weighting emerges from the recurrent interaction of multiple compartments within the dendritic tree, at the level of the input-output transfer function, the effective computation can be described in a feed-forward manner". Here, and and in the results section, the authors treat the computation of the neuron with dendrites as feedforward, despite its recurrency. On Page 9, para 1: "We hypothesize that the backpropagating action potential rate that codes for us* can influence dendritic synapses." Spatial properties through which the bAP impacts the dendritic tree, would need to be addressed with some recurrent interaction of multiple compartments. I recommend a discussion of recurrence as a limitation of the proposed framework and a need to address recurrence in future models that may explain bAP's role in setting the target input.*

The review correctly points out that correctly describing bAPs requires an extension of our formalism due to the recurrence this introduces. We mention this point now in the discussion as a potential extension.

(7) *2. Results:*

2.1. Opinion weighting in cortical neurons

2.1.1. **Clarity** *→ Page 3, para 1: "In the absence of stimuli, the 'cat neuron' has a low prior opinion. . . ". As a reader I was confused if the "cat neuron" corresponded to the bimodal (VA, see fig 2) input neuron. I suggest making this a little clearer.*

We have now added a reference to the respective panels, we hope this makes this clearer.

(8) *2.1.2.* **Clarity** *→ Fig. 2: Is the neuron in 2d and 2e different from the neuron 2c? If they are different, is the neuron in 2d/2e receiving input from the neuron 2c as the VA input? In addition, is the VA input the "cat neuron" or is the neuron in 2d/2e the "cat neuron"? I suggest making this a little clearer.*

We have now added additional explanation to make this clearer.

(9) *2.1.3.* **Clarity** *→ Fig. 2e: Is the opinion (which is labeled the "somatic membrane potential of the cat neuron" in page 3, para 1) interchangeable between E_d and u_d? I suggest making this a little clearer*

The trace in Fig. 2e represent the somatic membrane potential u_d of the "cat neuron" under three different

stimulation conditions, which is "driven" by the somatic reversal potential E_d. We hope this distinction becomes clearer in the revised manuscript.

(10) *2.2. The neuronal opinion code*

2.2.1. **Limitations/Scope***: Non-monotonic transfer functions → Page 6, para 2: "In our framework the somatic membrane potential is transformed into an instantaneous output rate... via some monotonically increasing transfer function..." It is fair to say in this particular framework this transfer function can be monotonically increasing. However, it is known that non-monotonic dendritic phenomena, such as dCaAPs (increasing excitatory input intensity can yield decreasing somatic depolarization) (Gidon et al., 2020), can result in very interesting dynamics that would fall outside of this framework. I recommend some treatment of these non-monotonic response phenomena in the discussion when addressing the list of phenomena that do fall within this framework. As a reviewer, I understand thorough treatment of this subject may fall outside of the scope of this work, however acknowledging these phenomena may be a good communication of the potential limitations of this analogy. Stimuli elicit neuron-specific opinions and increase the neuronal reliability*

First, we would like to clarify we assumed that the transfer function is driven by the somatic membrane potential, not the neuron's input. However, the reviewer correctly points out that biological phenomena exist which resist such a simple description. Since it is not essential for our model, we removed the reference to monotonically increasing transfer functions to avoid confusion.

(11) *2.2.2.* **Assumption/Justification***: Subtree Simplification → Page 6, para 4: This paragraph describes the opinion weighting calculation within the neuron with dendrites. In it, it assumes that there is one opinion per dendritic subtree. This assumes there cannot be 2 opinions on a single subtree, which removes the need to consider spatial effects on the interaction of 2 or more opinions within one subtree.*

2.2.2.1. I would like to see the justification for this simplifying assumption, given the phenomena of super-linearity (Polsky et al., 2004) from synaptic interactions within dendritic subtrees and synaptic clustering (Kastellakis & Poirazi, 2019) in general that may be important for the computations within the single neuron. Otherwise, I would like to see in the discussion a treatment of this simplifying discussion, as well as how it may or may not be a point of departure for future research and development of the proposed theory.

2.2.2.2. Page 8, para 2: In the section "Bayesian neural dynamics", the authors write that the models are "bidirectionally-coupled multi-compartment models with leaky integrator dynamics and conductance-based synaptic coupling under the assumption of fast dendritic responses." Here, it shows why inter-synaptic interactions are not considered in this framework's development.

2.2.2.3. Page 14, Eq 6: In the Methods, in the section "Equivalent somato-dendritic circuit", the authors give an equation for the dynamics of the somatic potential u_s, which is a linear sum of the soma compartment conductance and the dendritic conductances. The authors use this as a premise for the rest of their derivation for their framework. Treating entire dendritic subtrees as singular compartments simplifies away spatiotemporal relationships between synapses. It is fair to make simplifications like this, however as a reader I am looking for a justification for this abstraction, given the complexity of biological reality. I recommend treatment of this assumption in the discussion.

The reviewer correctly points out that we significantly simplify the complex spatial topology of a neuron. In our approach compartments represent logical subdivision of the dendritic tree without restrictive assumptions on ther spatial organization. We choose the logical subdivision to simply map individual likelihood functions and prior distributions to the dendritic tree. An extension to spatially extended morphologies and more complex dendritic interactions presents an interesting direction for future work,

as we mention in the discussion of the revised manuscript.

(12) *2.2.3.* **Clarity** *→ Page 7, para 2: "increasing stimulus reliability". As a reader, I was confused by the language of "reliability" here given that reliability of opinion is invoked earlier. I would like to see how the 'reliability of opinion' and 'reliability of the stimulus' relate, if at all. clarity*

We now give an example for features influencing "stimulus reliability" (intensity). We hope this makes this clearer.

(13) *2.3. Learning Bayes-optimal cue combinations*

2.3.1. **Expansion***: Cross-validation and Potential Overfitting → Fig. 7: The caption of this figure, states that each experiment consisted of 20,000 trials. After looking through the supporting information, I do not see figures showing how learning performance improved over time through loss or training curves, often used in machine learning literature. This raises my concern that the network model may be overfit to the target, which could increase the "average loss in %" in figure 7c derived from the test trials. Though not necessarily important for the figure in the main text of the paper, I would like to see data checking for potential overfitting in the supplementary information. If there is overfitting, perhaps the authors could consider an early-stopping policy using cross-validation when training the network model. The suggestions may result in determining if the bimodal cues, the visual cues only, or the tactile cues only could yield more efficient learning of the target.*

In contrast to machine learning approaches, we do not have a fixed dataset on which our model is trained. Rather, we add noise to a small collection of pairs of inputs and targets. Since this noise is different for *every* sample the network encounters during the training process, overfitting can be excluded.

(14) *3 Discussion*

3.1. **Claim/Scope***: Dendritic Computations → Page 12, para 3: "All of these computations could be put into our framework of opinion pooling, where the calculations on different dendrites are combined according to their reliabilities." As a reader, I'm wondering if the authors are leaving these computations as exercises for the reader. I am not sure if making this claim without necessarily spelling them out elsewhere in the paper is sufficient for the discussion section of this paper; however, I would be satisfied if it were made clear that demonstrating these computations is outside of the scope of the paper.*

We agree with the reviewer that such a claim requires at minimum a toy model, and thus have removed the corresponding paragraph from the revised manuscript.

(15) *3.2. Claim/Scope: Single Neurons to Populations → Page 13, para 3: "In our framework, to read out the reliability of a single neuron's opinion about the presence of its presynaptic feature, postsynaptic neurons either have to average across time or across a population of neurons that encode the same feature. Our single-neuron description of Bayesian inference is complementary to these population-based models." I am not sure if making this claim without necessarily spelling it out elsewhere in the paper is sufficient for the discussion section of this paper; however I would be satisfied if it were made clear that demonstrating this complementarity is outside of the scope of the paper.*

We agree with the reviewer and have weakened this claim.

(16) *3.3 In general, the authors could have given more attention to assumptions and limitations of their framework and used this point of discussion to suggest future work.*

We agree and point these out where possible in the constraints of an accessible manuscript.

Reply to comments of reviewer #2

(1) *I believe I understand Bayesian inference pretty well, especially in the Gaussian setting, but I did not understand what's going on in this paper until I sat down and figured it out on my own. And even then I'm guessing I don't have the full story. Following is what I think the authors did.*

We thank the reviewer for their time and patience in reviewing our manuscript. We appreciate their feedback and hope the revised version is more accessible. Below we have addressed all issues raised by the reviewer in a point-to-point reply.

(2) *In the end, it's kind of a simple story. Sort of. Let's start with a review of Bayesian inference when all distributions are Gaussian. If we have a set of observations, E_i, with variance σ_i^2, and the prior is Normal(x_0, σ_0^2), then, defining $g_i = 1/\sigma_i^2$, the posterior is Gaussian with: mean $= (\sum_i g_i E_i)/(\sum_i g_i) 1/variance = \sum_i g_i$ where the sum starts at $i = 0$ (to take into account the prior). So if g_i is conductance and E_i is reversal potential, the mean looks a lot like the mean voltage in a conductance based model (so long as the neuron doesn't spike). But reversal potentials mainly come in two flavors: around $0mV$ for excitatory input and around $-80mV$ for inhibitory input. So how do we get an in-between membrane potential? From a weighted sum of excitatory and inhibitory inputs. The relative weights give the mean reversal potential; to get the overall strength you have to match the conductance (via the above equation).*

This is correct and describes the basis for neuron dynamics in our approach.

(3) *All this has to be learned, of course, and the authors write down a learning rule (Eq. 5). It's reasonable, but they don't derive it. I looked in Methods, but got a bit lost because I couldn't tell the difference between a scalar, vector and matrix, so I gave up. Without going through the details, though, it's hard to tell if this makes sense, especially since the firing rate distribution is going to be constantly changing. And to learn they need samples from the true posterior – it's totally unclear where that would come from. (The authors say backpropagating action potentials, but that doesn't really help, because then the soma has to know the posterior.)*

What's really needed is a cost function one can wrap one's head around, like performance on a task, rather then the KL divergence. With the right cost function and problem setup, everything would be easy to derive.

We regret that this did not became clear from our manuscript. Indeed the learning rule is derived from a loss function quantifying the divergence between a "target distribution", e.g., the true posterior, and the represented somatic posterior. This divergence determines "the performance on a task" (KL large → bad performance, KL small → good performance), however the choice of the target distribution depends on the task and is up to the modeler. For the sake of space we have not included the derivation in the main manuscript, but a brief derivation in Methods "Weight dynamics" and a detailed derivation in the Supplements. In the derivation we also explain why we don't need access to the full target distribution, but rather to samples from the distribution. This is equivalent to the typical "supervised learning" setting. We have added a comment in the revised manuscript to point this out.

(4) *On top of that, the scheme requires the dendritic time constant to be much faster than the somatic one. Which it isn't; they're about the same. Does this kill the idea?*

The dendritic time constants are indeed smaller than the somatic one, contrary to the reviewer's suggestion (e.g., Wybo, W. A., Torben-Nielsen, B., Nevian, T., & Gewaltig, M. O. (2019). Electrical compartmentalization in neurons. Cell reports, 26(7), 1759-1773.). In general, however, this assumption is a merely

helpful ingredient that simplifies the mathematical treatment (essentially, we don't have to wait for dendritic transients to settle), but in principle the theory can also be extended to slow dendritic responses, which is beyond the scope of this manuscript.

(5) *So my take is: it's possible that the authors have done something sensible, but their explanation was so inscrutable to me that I couldn't evaluate it. In my youth I probably would have gone through Methods and SI and figured it out, but that's really not my job. If the other reviewers are happy, I'm certainly OK with this being published, although my predictions is that very few people will ever understand what they did. However, it would be nice if they wrote the paper clearly enough that it could be easily understood by your run-of-the mill theorist with a decent math background. I don't think it is now. (Or maybe I just need to be a lot smarter – don't want to rule that out.)*

We thank the reviewer for their refreshingly honest review. We hope that the revised manuscript is significantly more accessible and would be understandable to a wider audience.

Reply to comments of reviewer # 3

(1) *The current study investigates the Bayesian computation in a single conductance-based neuron, where the membrane potential represents the posterior mean, and the conductance represents the posterior precision. In this way of representation, the single neuron dynamics can implement the Bayesian computation well. The authors also considered some tasks to demonstrate the performance of the model. Overall, I feel this work provide new insight onto Bayesian computation on single neurons, and I like the geometric interpretation of the Bayesian computation (Fig. 3).*

We thank the reviewer for their positive feedback and constructive criticism. Below we have addressed all issues raised by the reviewer in a point-to-point reply.

(2) *Major: The current model assumes that each dendritic compartment has an associated preferred feature (text below Eq. 1). Considering a single only has finite number of dendritic compartments, I am wondering how a neuron could represent and conduct the computation of the distribution over continuous variables? Does this require an idealized neuron with infinite number of dendritic compartments? If I misunderstood, how the digital to analog conversion can be done at single neuron level? Some discussions would be helpful.*

We agree with the reviewer that the phrasing in our original manuscript was somewhat confusing. We have now tried to make it more accessible and hope that the revised version makes these points clearer.

(3) *For simulations in Fig. 7: the text mentions that the uncertainty in the sensory input is modeled by different levels of additive noise. On the other hand, the current framework the likelihood uncertainty is represented by conductance in a dendritic compartment which is proportional to input firing rate, which means the sensory input firing rate encodes the input uncertainty. Therefore, I am puzzled without altering the input firing rate (with the same conductance) but just changing the input additive noise, how the single neuron could do the Bayesian computation optimally?*

The reviewer points out an essential feature of our approach: we rely on the presence of two types of conductances (excitatory and inhibitory) which encode two parameters (mean and variance). Furthermore, it is important to note that the *total* conductance codes for reliability Thus one can increase the mean without changing the variance or the variance without the mean as required by Bayesian inference.

(4) *Deneve, Neural Computation 2008a,b also studied the Bayesian computation in single neurons which is quite relevant to the current study. It might be good to compare the current work with Deneve's.*

Deneve's work on Bayesian single neurons is very interesting, but it is difficult, at the moment, to compare it to our approach since they consider temporal spiking codes, while our framework is fundamentally rooted in a rate-based view of neuronal computation.

(5) *Minor: The text above Eq. 15: is it should be the inverse of variance?*

Yes, we thank the reviewer for pointing this out.

(6) *The text after Eq. 17: the Langevin sampling from the posterior seems to be over claimed. Implementing the Langevin sampling is not merely injecting noise into the gradient ascent dynamics on the posterior, but it has a stronger requirement that the drift and fluctuations terms share the same factor. The current manuscript doesn't provide any support about this.*

We agree with the reviewer and have accordingly weakened this claim.
