# Supplementary material for: Conductance-based dendrites perform Bayes-optimal cue integration
Source: PLoS Comput Biol. 2024 Jun 12;20(6):e1012047. doi: 10.1371/journal.pcbi.1012047 (PMC11168673; doi:10.1371/journal.pcbi.1012047)
Supplement: Attachment — Submitted filename: reply_letter.pdf [file pcbi.1012047.s004.pdf]

Jakob Jordan
Department of Physiology
University of Bern
Bühlplatz 5, CH-3012 Bern
Switzerland
email: jakob.jordan@unibe.ch

February 6, 2024

Robert E. Kass
Editor, PLOS Computational Biology
Thomas Serre
Editor, PLOS Computational Biology

Resubmission of manuscript PCOMPBIOL-D-22-01785

Dear Robert E. Kass, dear Thomas Serre,

thank you for your reply and for your feedback. We are delighted to see that at least two of the reviewers are now happy with the manuscript.

We believe that the position of Reviewer 2 follows mainly from several misunderstandings that we think are easy to alleviate, with reference to the manuscript in its current form. Below, we address these point by point.

We have also slightly amended the manuscript in the process, but we hope that with the clarifications below are enough to convince Reviewer 2 that the manuscript either already addresses their critique directly and specifically, or that it - fortunately - does not apply.

Best regards,
Jakob Jordan (on behalf of all authors)

encl: - reply to reviewer #1
- reply to reviewer #2
- reply to reviewer #3
- revised manuscript with and without highlighted changes

Reply to comments of reviewer #1

(1) *Thank you very much for responding to my comments and suggestions. I found all responses acceptable and that the revised version of the manuscript addresses my previous concerns. The clarity of the manuscript has improved. (5), (6), (11) are sufficiently addressed in the discussion. (7), (8), (9), (12) are sufficiently addressed in the results. The language in question for (10) and (14) has been removed. I agree with the authors for (13) that this is not an issue. The claims, scope and limitations suggested and highlighted are effectively addressed in (14) and (15).*

We again thank you for your feedback and your evaluation!

Reply to comments of reviewer #2

(1) *In my previous review, my main complaint was that the paper was very hard to make sense of. That hasn't changed a lot; the paper is still very hard to make sense of. This time, however, I took a closer look at Methods (the only way to figure out what was actually going on). I think I sort of know what they're doing, but now things don't exactly make sense to me.*

So here's a quick summary: information about the true membrane potential of a neuron come from "dendrites" (by which they mean dendritic branches?). Because they're using conductance-based synapses, each dendrite encodes the mean and variance of a Gaussian distribution. That mean and variance is combined optimally by the post-synaptic neuron.

So here's what I find problematic:

1. After digging through supplementary materials, I found that for the excitatory and inhibitory conductances, the authors use

$$g_i^E = W_i^E r$$
$$g_i^I = W_i^I r$$

where r is the (global?) firing rate. At least I think that's what they use; I had to infer that from Eq. 13 of supplementary materials (I couldn't find it explicitly stated anywhere else, but maybe I missed it?). But this isn't what happens on real neurons; instead, the membrane potential on a dendrite is driven by many different inputs, from many different neurons. So it should be

$$g_i^E = \sum_j W_{ij}^E r_j^E$$
$$g_i^I = \sum_j W_{ij}^I r_j^I$$

We greatly appreciate the reviewer's willingness to carefully go through the mathematical details, including those in the supplement. In this particular case, this might not have been necessary - the above equations are given in the main manuscript, directly below Eq. 1: "The excitatory and inhibitory conductances are the product of the synaptic weights times the presynaptic firing rates, $g^{E/I} = W^{E/I} r$." As also stated in this sentence, it is the presynaptic firing rates that are denoted by r.

The sentence before that states: "The sum of these three conductances $g^d = g^E + g^I + g^L$ represents the local membrane conductance, which excludes the coupling to other compartments." So here we are talking about a small dendritic branch or compartment, for which we can assume only one presynaptic input rate r. Thus, and to avoid unnecessarily complicating the notation at this point, we chose to omit the presynaptic indices.

Further on, we choose to sum over compartmental indices i, as described in the immediately following paragraph and in Eq. 3. In other words, the reviewer's equations are absolutely correct, but we simply prefer a different, more sleek notation, especially considering the complexity of later equations in the manuscript.

In an attempt to be even more explicit, we have added some further explanatory text around Eq. 1. We have also used bold variables for vectors and matrices to more clearly distinguish them from scalars (in

addition to the latter having an index). We have further modified our notation for the energy in the Methods section (Eq. 9, 16), to more clearly distinguish it from the effective reversal potential.

(2) *This means the conductances – and thus the mean and variance of the likelihood on dendrite i – is determined not just by the weights, but also by the firing rates of different populations of neurons. That's fine, but it needs a much more sophisticated learning rule than the one given in the paper. This is a central issue that needs to be addressed.*

This must be another misunderstanding. The reviewer is absolutely correct with their first statement, and our rule addresses this exact scenario. Multiple equations in the manuscript make that abundantly clear - this is what the index i (and sums over i) is used for - for example in the neuron dynamics (Eq. 3), but also in the learning rule itself (Eq. 4). Still, the learning rule remains local, as discussed explicitly below Eq. 4.

(3) *2. Given the setup, I was expecting a learning rule that would ensure that each dendritic branch carried the correct mean and variance. But that's not what the learning rule, Eq. 4, seems to do. For instance, take the case in which u_s^* is fixed. In that case, the learning rule would first drive the difference in the excitatory and inhibitory weights so that $\bar{E}_s = u_s^*$, then drive magnitude of both to infinity (to make $\bar{g}_s$ as large as possible). Moreover, the weights would be infinity independent of the reliability of the different dendrites. Which seems odd to me.*

On the contrary, that is exactly what should happen in a rigorous Bayesian setting! It is quite satisfactory to see the reviewer deduce that this is what our learning rule predicts.

If the target membrane potential u_s^* is fixed, it has, by definition, infinite precision. To overrule a prior with non-zero precision (finite variance), Bayes requires infinitely strong evidence. Since in our setting the prior does have non-zero precision (non-zero g^L), synaptic weights on the dendrites need to increase to infinity to construct the infinitely precise posterior given by the fixed u_s^*. Note also that even in this pathological scenario, i.e., if weights diverge, the relative ratio of the different dendritic branches remains finite and represents their relative reliabilities. And of course for less pathological cases, such infinite weights can not occur.

(4) *At least that's what the learning rule says. But on page 9 the authors say:*

"To illustrate these learning principles we consider a toy example in which a neuron receives input via two different input channels with different noise amplitudes. Initially neither the average somatic membrane potential, nor its variance match the the parameters of the target distribution (Fig. 6a, left). Over the course of learning, the ratio of excitatory to inhibitory weights increases to allow the average somatic membrane potential to match the average target potential and the total strength of both excitatory and inhibitory inputs increases to match the inverse of the total somatic conductance to the variance of the targets (Fig. 6a, right; b1)."

This implies that they can indeed learn the correct mean and variance. But I simply could not figure out how.

Yes, it is true that our plasticity rule can learn the correct mean and variance, as also discussed by the reviewer for the rather extreme case above. Why the rule does this follows directly from its derivation: it performs gradient descent on the KL divergence between posterior and target distribution. This is explicitly stated above Eq. 4 and derived in detail in the Methods (Section "Weight dynamics", p. 16, in particular Eq. 17 and 18).

(5) *And, as an aside, the authors say "We hypothesize that the backpropagating action potential rate that codes for* u_s^* *can influence dendritic synapses [30]." But the backpropagating action potential rate is just the firing rate of the neuron. How would the neuron know how to set its firing rate to match* u_s^**?*

The argument for how u_s^* can ultimately reach the synapse is provided in the given reference [30]. In brief, in the limit of weak nudging, the neuron's firing rate is pulled slightly in the direction of u_s^*, thus backpropagating a useful proxy of u_s^*. As the nudging increases, the backpropagated activity grows increasingly closer to u_s^* itself. Since the given reference [30] provides such a direct answer to the reviewer's question, we appeal to the usual academic practice of referencing literature without reiterating its content.

(6) *3. The authors assume that different dendrites are independent. However, we now know that that's a bad idea, and, at least in some circumstances, correlations totally dominate when it comes to information (Information-limiting correlations, Moreno-Bote, et al., Nature Neurosci. 17:1410-1417 2014). So this seems like a seriously problematic assumption.*

We do not assume such independence anywhere in the derivation of neuron and synapse dynamics (Eq. 3 and 4). However, we do assume conditional independence of dendrites given the somatic potential, which is a different thing and in no way precludes correlations between dendrites. We suspect that this might be the source of the confusion, but this assumption is spelled out explicitly between Eq. 11 and 12 in the Methods.

(7) *4. Finally, it's not clear why the brain cares about* $p(u_s^*)$*. Doesn't it want a probability distribution over task-relevant variables? I'm not saying it doesn't want* $p(u_s^*)$*, I'm just saying it's not obvious to me why it does. The authors need to motivate this a little better.*

We agree that the brain doesn't need to care directly about p(u*). But to achieve target distributions over task-relevant variables, it is sensible to assume (and universally accepted in the field as a default hypothesis) that learning implies the formation of such explicit or implicit targets p(u*) for the constituent neurons of the brain. We address this in the 1st paragraph of the discussion: "The targets may themselves be formed by peri-somatic input from other modalities, or by more informed predictive input from other cortical areas."

(8) *The bottom line is slightly worse than last time: I now have several reservations about the paper. However, I strongly suspect that I didn't fully understand it (which in itself is a problem), so possibly the reservations are easily addressed.*

We hope that our additional explanations in this response help alleviate the reviewer's reservations.

Reply to comments of reviewer # 3

(1) *I am overall satisfied about the revisions made by the authors, while I suggest the authors make below revisions.*

Please refrain from using the Langevin sampling below Eq. 16 because the current method only outputs the MAP rather than the whole posterior distribution. An alternative might be "... using Langevin dynamics to find the MAP solution...". In addition, the authors may be interested in a nonlinear circuit implementation of Langevin sampling https://doi.org/10.1101/2020.07.20.212126

We accept the reviewer's suggestion and have modified the text accordingly (below Eq. 16).

(2) *I admit that the representation in current study is quite distinct with Deneve 2008. But it would be quite beneficial for readers to compare different studies if the author could discuss this briefly in Discussion.*

We agree that our study is very different from Deneve 2008. Because these significant distinctions across multiple aspects of the model are so pronounced (neuron and synapse dynamics, studied tasks, comparison to data etc.), we fear that a useful comparison would be difficult to set up and might only confuse the reader.
